# Polypyrrole Nanomaterials: Structure, Preparation and Application

**DOI:** 10.3390/polym14235139

**Published:** 2022-11-25

**Authors:** Lu Hao, Changyi Dong, Lifeng Zhang, Kaiming Zhu, Demei Yu

**Affiliations:** 1 School of Chemistry, Xi’an Jiaotong University, Xi’an 710049, China; 2 State Key Laboratory of Electrical Insulation and Power Equipments, Xi’an Jiaotong University, Xi’an 710049, China; 3 MOE Key Laboratory for Non-Equilibrium Synthesis and Modulation of Condensed Matter, Xi’an Jiaotong University, Xi’an 710049, China; 4 Joint School of Nanoscience and Nanoengineering, North Carolina Agricultural and Technical State University, Greensboro, NC 27401, USA

**Keywords:** polypyrrole, nanostructures, synthetic methods, properties, applications

## Abstract

In the past decade, nanostructured polypyrrole (PPy) has been widely studied because of its many specific properties, which have obvious advantages over bulk-structured PPy. This review outlines the main structures, preparation methods, physicochemical properties, potential applications, and future prospects of PPy nanomaterials. The preparation approaches include the soft micellar template method, hard physical template method and templateless method. Due to their excellent electrical conductivity, biocompatibility, environmental stability and reversible redox properties, PPy nanomaterials have potential applications in the fields of energy storage, biomedicine, sensors, adsorption and impurity removal, electromagnetic shielding, and corrosion resistant. Finally, the current difficulties and future opportunities in this research area are discussed.

## 1. Introduction

Traditional polymer materials have good insulation properties and are one of the most used materials in the world today. However, in 1977, A. J. Heeger, A. J. MacDiarmid and H. Shirakawa synthesized a new type of polymer material. The conductivity of polyacetylene doped with iodine was significantly improved to 10^3^ S cm^−1^ [1]. Subsequently, a series of polymers with similar properties such as polyaniline, polythiophene, and polypyrrole were discovered, which largely motivated the development of conductive polymers (CPs). PPy has attracted much attention because of its advantages, namely simple preparation, nontoxicity, good stability, excellent mechanical properties, and high conductivity, which may make it the next conductive polymer that can be industrially produced and applied in many fields. However, conventional PPy with an amorphous phase has poor solubility and mechanical ductility, resulting in insolubility and infusion in most organic solvents and making it difficult process into specific shapes. More importantly, traditional bulk PPy lacks good electrical, optical, and biological properties due to its amorphous morphology, so the structure and size must be tuned to achieve optimal performance. Benefiting from the well-defined nanotopography and larger surface area, nano-PPy has peculiar electrochemical activity, better optical properties, and excellent biocompatibility compared with bulk PPy [2]. As nano-PPy can be fabricated into a variety of nanostructures ranging from zero-dimensional nanoparticles, one-dimensional nanotubes/nanowires and two-dimensional nanosheets, to three-dimensional nanonetworks, so an in-depth comprehension of preparation strategy, and morphology control, as well as the relationship between structure and performance, is essential for promoting further research, as well as the development of high-performance applications of PPy nanomaterials [3,4,5,6].

Taking this as an opportunity, this paper outlines the research progress of PPy nanomaterials since 2010, focusing mainly on three aspects: structure and properties, preparation, and application of PPy nanomaterials. The structure of PPy nanomaterials includes four types: PPy nanoparticles, PPy nanotubes, PPy nanowires, and PPy nanosheet. The preparation of PPy nanomaterials include three main methods: the soft micellar template method, the hard physical template method, and the templateless method. PPy nanomaterials have potential applications in the fields of energy storage, biomedicine, sensors, adsorption and impurity removal, electromagnetic shielding, and corrosion resistance. The applications of PPy nanomaterials are concentrated in the following areas: energy storage, biomedicine, sensors, adsorption and impurity removal, electromagnetic shielding, solid-phase extraction, and actuators. Finally, the current difficulties and future opportunities in this research area are discussed.

## 2. Types of PPy Nanomaterials

Classified by structure, there are four main types of common PPy nanomaterials: nanoparticles, nanotubes, nanowires and nanosheets.

### 2.1. PPy Nanoparticles

**Figure 1:**
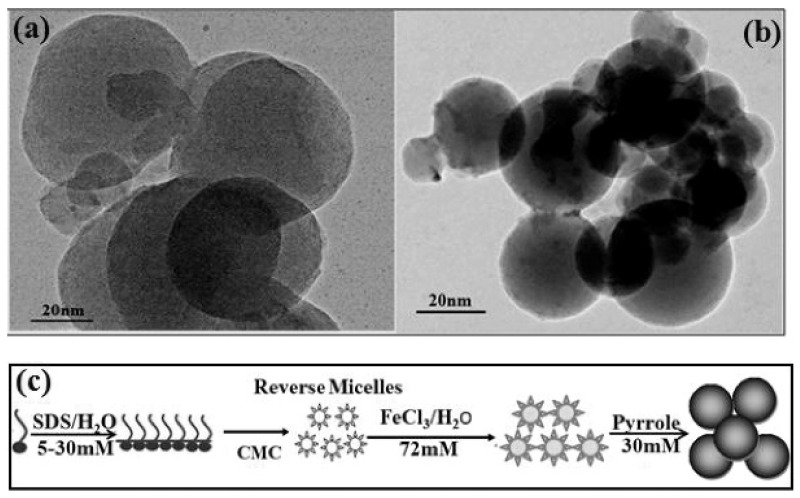
(**a**,**b**) TEM images of PPy NPs and (**c**) schematic representation of the preparation of PPy NPs [11]. Copyright 2014 Journal of Applied Physics.

PPy nanoparticles are the most common type of PPy nanomaterial. The formation of granular PPy often requires the participation of surfactants. Surfactants usually form micelles in solution, which not only act as templates but also reduce the active energy of the polymer surface and make it stable. Pure PPy has poor conductivity, and dopants need to be added to improve its conductivity. The formation and properties of PPy nanoparticles (NPs) are also affected by surfactants and dopants [7,8,9,10,11,12,13,14,15,16,17,18,19]. Rawal et al. [11] prepared PPy NPs at various concentrations of sodium dodecyl sulfate (SDS) and investigated the mechanism of charge transport in PPy NPs, as shown in Figure 1. The conductivity of the prepared NPs increases from 3 to 22 S cm^−1^ when the surfactant is used. Minisy et al. [19] used chemical oxidative polymerization to increase the conductivity of PPy from 1–5 S cm^−1^ to 84 S cm^−1^ by regulating the concentration of the dopant methyl red salt. When the reaction temperature was lowered, the conductivity of PPy was further improved.

The formation of PPy NPs was also influenced by oxidants and other conditions [20,21,22,23,24]. By introducing 2, 4-diaminodiphenylamine as an initiator into the reaction mixture, Liao et al. [20] synthesized water-dispersed PPy nanospheres with high yield without any template. It was found that the morphology and size of the prepared PPy nanospheres were affected by the concentration of initiator, oxidizer and acid. Among them, spherical PPy nanostructures with smaller diameters can be obtained when smaller acids (the size of anions) are used. Hong et al. [23] explored a facile method to synthesize PPy NPs with diameters of 20–60 nm. It was found that PPy NPs with a narrow size distribution can be easily manufactured by reasonably adjusting the hydrodynamic radius, turning radius, shape factor and viscosity.

In general, the preparation of PPy NPs uses ferric chloride, ammonium persulfate and hydrogen peroxide as oxidants. Compared with the other two oxidants, the byproduct of hydrogen peroxide is only water, which is cleaner and environmentally friendly. However, the reaction rate of polymerization was slow when using hydrogen peroxide as an oxidant, which hinders its application [25]. To solve this problem, our group [26,27] prepared uniform PPy and its derivative NPs using H_2_O_2_ with the aid of UV radiation in the existence of polyvinylpyrrolidone with high efficiency. The involvement of UV light accelerated the polymerization of pyrrole without introducing impurities into the product.

Particles of different sizes often have different properties. The controllable preparation of PPy NPs is of great significance. Hong et al. [28] prepared three monodisperse PPy NPs with particle sizes of 20 nm, 60 nm, and 100 nm by dispersion polymerization. It was found that the incorporation of 20 nm PPy into organic bistable memory devices enables stable multistage switching with high on-off ratios. Jang et al. [29] prepared five monodisperse PPy NPs with different diameters, ranging from 20 to 100 nm, in the presence of Polyvinyl alcohol (PVA) to assess scale-dependent cytotoxicity. It was found that PPy NPs with an average diameter of 60 nm had the highest adverse reactions to the test cells. Zhou et al. [30] synthesized PPy nanoparticles with precisely controllable particle size by chemical oxidative polymerization, and systematically investigated the relationship between size and electrochemical capacitance properties. It was found that the capacitive properties of PPy nanomaterials are influenced by the synergistic effect of particle size, surface area, and charge carriers, and optimizing the size of the polymer material to 80 nm can significantly improve the performance. Kwon et al. [31] prepared PPy NPs with diameters of 20, 60 and 100 nm with the aid of the PVA/FeCl_3_ system. The conductivity and specific surface area of the PPy NPs decreased with the increasing of particle diameter, while the sensitivity of the gas sensors prepared based on the PPy nanoparticles increased with increasing particle diameter.

In addition to spherical particles, researchers have also prepared other nanoparticles with different morphologies. Yang et al. [32] used porous hollow gold nanocages as templates to grow PPy layers with uniform thickness on the inner and outer surfaces of gold nanocages by chemical oxidation and then selectively removed the gold nanocages through etching to form a double-walled “back” shaped PPy shell. Lee et al. [33] synthesized urchin-like PPy nanoparticles with different diameters by using a dual-nozzle approach, thus fabricating a sensor with extremely high selectivity to NH_3_. Using polymethyl methacrylate nanospheres and polystyrene hollow spheres with porous surfaces as hard templates, Su et al. [34] and Xia et al. [35] obtained monolayer PPy hollow spheres and the unique structure of PPy double-shell hollow particles, respectively. Qiao et al. [36] synthesized bowl-shaped PPy particles by adding N-methyl pyrrole into an iodine-containing pyrrole solution. Table 1 summarizes the relevant references for classifying the synthetic conditions, size and conductivity of PPy nanoparticles.

### 2.2. PPy Nanotubes

**Figure 2:**
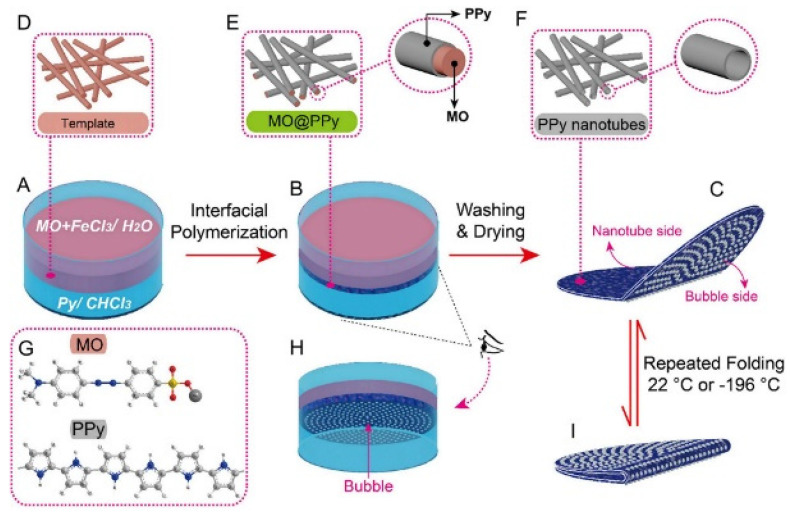
Diagram of the template-assisted interfacial polymerization (TIP) of a flexible PPy membrane (PPy-N). (**A**) Beginning of TIP (**B**) End of TIP (**C**) Final PPy-N membrane obtained after washing and drying. (**D**) Nanorod template structure formed by FeCl_3_ and MO. (**E**) MO@PPy nanorod structure on the water side of the membrane. (**F**) PPy nanotube structure following removal of templates. (**G**) MO and PPy molecular structure. (**H**) Bubbles on the chloroform side. (**I**) Final PPy-N membrane is flexible [52]. Copyright 2017 ACS Nano.

Due to its highly ordered structure, large specific surface area and superior carrier transport capacity, compared with corresponding bulk materials, PPy nanotubes (NTs) have significant advantages, such as larger surface area, excellent mechanical properties and high catalytic activity, so their application functions are extensive. In 1990, Martin et al. first prepared PPy nanotubes using the template method. The diameter and length of the tubes can be adjusted by changing the characteristics of the template film. The electronic conductivity of the prepared polymer nanotubes has also been significantly improved [37]. Using methyl orange (MO) as the dopant and FeCl_3_ as the oxidant, Yang et al. [38] prepared PPy NTs in large quantities by simple stirring at room temperature without any template for the first time. Because of its excellent characteristics, this method has been widely used. However, the formation mechanism and influencing factors of PPy NTs have also been widely studied by other researchers [39,40,41,42,43,44,45,46,47,48,49,50,51,52]. Mao et al. [52] obtained the film composed of PPy NTs by template assisted interfacial polymerization, as shown in Figure 2. In addition, Kumar’s team [53,54,55,56] used the MO-FeCl_3_ self-degradation micellar to support the growth of PPy NTs with the assistance of cetyltrimethyl ammonium bromide (CTAB) and found that the diameter of tubes decreased with the increase in CTAB concentration.

In addition, other methods of preparing PPy NTs are also constantly being reported, such as the template free method, the soft template method using other surfactants and the hard template method mainly using metal oxides as sacrificial templates [57,58,59,60,61,62,63,64]. Wei et al. [62] prepared PPy arrays using the templateless electrochemical method, which can easily be tuned between high adherent hydrophobic NTs and low adherent hydrophilic nanotips using an electrochemical redox process to dynamically attach and separate mesenchymal stem cells at the nanoscale. Trchova et al. [63] used resonance Raman spectroscopy to propose and establish correlations between conductivity, surface area, the ratio of ordered and disordered PPy phases on the surface and interior of the nanostructures. Minisy et al. [64] prepared PPy nanotubes in the presence of the cationic dye saffron and phenol saffron. Saffron supported the one-dimensional growth of PPy, and the PPy spheres became nanorods and later nanotubes as the concentration of saffron in the solution increased. In the case of the phenol saffron dye, the resulting PPy nanotubes were very thick and always accompanied by particles.

### 2.3. PPy Nanowires

**Figure 3:**
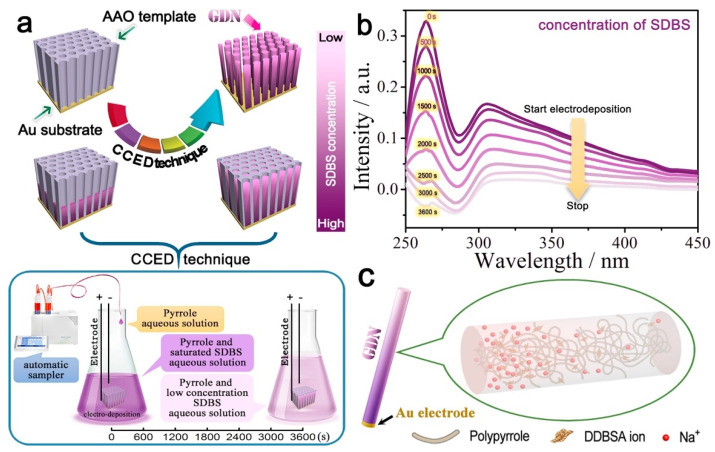
(**a**) Schematic illustration of the template growth of GDNa, (**b**) Ultraviolet spectrometry at different stages of electrodeposition solution, (**c**) Model diagram single GDNw [75]. Copyright 2018 Nano Energy.

PPy nanowires (NWs) not only have some of the excellent properties of conducting polymers, but also some of the unique properties of nanomaterials. There are many ways to synthesize PPy NWs [65,66,67,68,69,70], due to their excellent electrical properties and good biocompatibility, they have potential applications in many fields [71,72,73,74,75,76]. Nie et al. [75] created a “wet electric” nanogenerator based on gradient-doped PPy NWs using concentration-controlled electrodeposition (CCED) technology, as shown in Figure 3. The special component and structure of gradient-doped PPy NWs enable them to have a large surface area and one-dimensional transport nanochannels, which can greatly promote the diffusion of water molecules to produce free charged ions as free carriers.

Sun et al. [76] prepared proton-doped PPy NWs using ammonium persulfate and pyrrole monomers with different proton sources. The doping effect is the decisive factor in improving the electromagnetic absorption performance of PPy NWs based composites. Only the proton-doped PPy NWs with a load of 10 wt% can realize an EA bandwidth of 6.72 GHz (2.44 mm thickness), and the reflection loss value is less than −10 dB.

**Figure 4:**
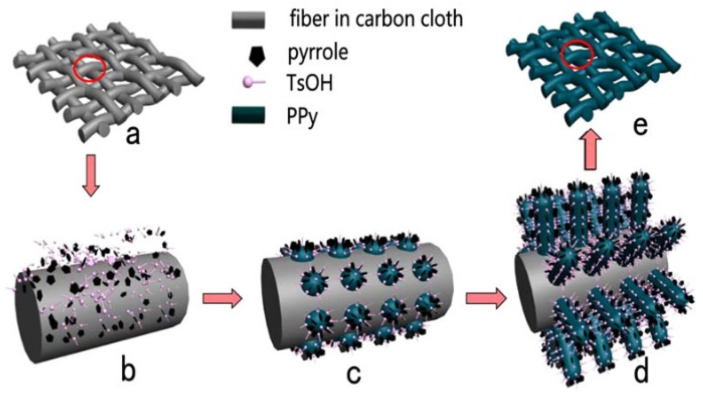
Growth of PPy NWAs on carbon cloth: (**a**) carbon cloth; (**b**) absorption of pyrrole and TsOH on the surface of a carbon fiber in carbon cloth; (**c**) nucleation of PPy on the surface of a carbon fiber; (**d**) PPy NWAs on the surface of a carbon fiber; (**e**) carbon cloth with PPy NWAs [82]. Copyright 2015 ACS Applied Materials and Interfaces.

In recent years, PPy NWs arrays have received attention from researchers and have broad application prospects in the field of supercapacitors and sensors [77,78,79,80,81,82,83,84]. Using oxidative polymerization in air, Kim et al. [78] obtained stretched monomeric menisci by pulling on a micropipette containing a Py solution. The radius of the wire thus generated is precisely controlled to 50 nm by adjusting the pulling speed. Huang et al. [82] constructed a 3D conductive layered structure through electrochemically fabricating ordered PPy nanowire arrays on the surface of carbon fibers, as shown in Figure 4. Xing et al. [84] reported a simple method for preparing antibacterial peptide modified PPy NW array electrode (PNW-AMP). The PNW-AMP electrode exhibits excellent oxidation-reduction and low interface resistance characteristics, and can eliminate bacterial adhesion in the microbial microenvironment while maintaining electrochemical stability for a long time.

In addition, as a special structure of NWs, PPy nanobelts have also attracted the attention of researchers [85,86,87,88]. Chi’s team [85,86] prepared 1D conducting polymer nanobelts with an average width of 50 nm and investigated the conductivities of individual PPy nanobelts by using conductive atomic force microscopy.

### 2.4. PPy Nanosheets

**Figure 5:**
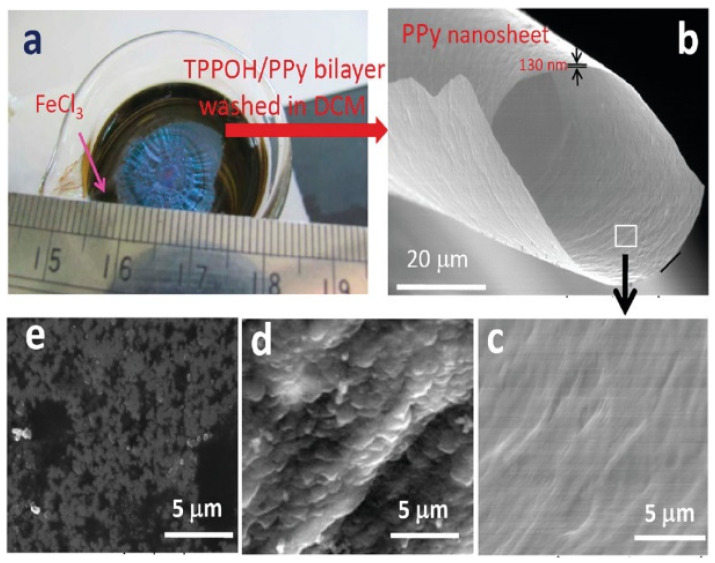
(**a**) Photograph of the TPPOH/PPy bilayer formed at the air/FeCl_3_ interface film, (**b**) SEM images: PPy-2 film, (**c**) magnified image of PPy-2 film, (**d**) J-aggregate film of TPPOH, and (**e**) PPy-1 films that are formed without TPPOH [91]. Copyright 2011 Macromolecules.

PPy nanosheet is one of the PPy nanomaterials for which there are few studies. The typical method of synthesizing nanosheets is the organization and polymerization on the interface, such as Langmuir-Blodgett film, solution-phase synthesis and chemical vapor deposition (CVD). A typical example of a nanosheet is graphene, which is the thinnest two-dimensional material. In addition, metal nanosheets have also been obtained by reducing metal precursors such as palladium, rhodium or gold in solution. In recent years, the synthesis and applications of PPy nanosheets have also been reported by researchers [89,90,91,92,93,94]. Jha et al. [91] prepared free-standing PPy nanosheets using a one-pot method by dropping a porphyrin derivative (TPPOH) and pyrrole into an FeCl_3_ solution, as shown in Figure 5. The results show that TPPOH rapidly forms a J-aggregate film at the air/FeCl_3_ interface, which can provide an in-situ template for the growth of PPy nanosheets.

In addition to the several nanostructures mentioned above, researchers continue to prepare PPy nanomaterials with new shapes [95,96,97,98,99,100,101,102,103]. Liu et al. [95] synthesized 2-D PPy nanoclips with diameters ranging from 50 to 70 nm using an oxidative template consisting of cetrimonium cations and peroxydisulfate anions. Bao et al. [98] fabricated a biomimetic hydrogel/nanoporous PPy asymmetric heteromembrane with electrical/pH-responsive 3D micro/nanoscale ion channels. Since the charge density in the membrane can be regulated by electrical stimulation and pH stimulation, the ionic rectification of the membrane shows responsive properties.

## 3. Preparation of PPy Nanomaterials

Compared with conventional PPy, nano-PPy shows better conductivity, higher specific surface area, shorter ion migration distance and good electrochemical activity. The preparation methods for nano-PPy include the soft micellar template method, hard physical template method and templateless method.

### 3.1. Soft Micellar Template Method

The soft micellar template method, also called the self-assembly method, generally uses the interaction between the hydrophobic group and the hydrophilic group in the amphiphilic molecule to form a specific micelle in the solvent, and the monomer forms a specific morphology inside or on the surface of the micelle. These nanomaterials are usually prepared by microemulsion polymerization, which can obtain polymer nanomaterials with controllable sizes. The soft template method is usually used to prepare PPy NPs and NTs materials. The structure and concentration of monomers and surfactants are the key factors for controlling product morphology parameters.

Using CTAB as a template and 1,5-naphthalene disulfonic acid (1,5-NDA) as a dopant, Han et al. [104] synthesized a hierarchical nanostructured PPy in an aqueous solution with potential applications in the field of supercapacitor materials. The concentrations of pyrrole and CTAB, as well as the rate of polymerization, had an obvious effect on the formation of the hierarchical structures. Northcutt et al. [105] proposed a biotemplate method for the three-dimensional surface modification of dodecylbenzenesulfonate (DBS)-doped PPy membranes. The results show that the existence of a biotemplate enables the bulk of the polymer to create morphologies with a high specific surface area, and raises the surface area of interface. Due to the higher ionic current, the three-dimensional PPy film has a higher specific capacitance than that of the planar PPy film. Chen’s team [106] synthesized nanoscale PPy particles by a soft templating method with the aid of Triton X100 micelles. The surface acoustic wave sensor containing the nano-PPy particles can detect acetone. Furthermore, using CTAB as a soft template, his team synthesized ultralong interconnected PPy NWs via an organic phase (pyrrole)/aqueous phase (oxidant) interfacial reaction [107]. The size and morphology of the prepared PPy can be selectively modulated by changing the concentration of CTAB. In addition, the CTAB concentration plays an important factor in enhancing the electrochemical performance of the prepared PPy NWs.

### 3.2. Hard Physical Template Method

The hard physical template method uses the material with a special inner or outer surface as the template, fills the polymer monomer into the template, and synthesizes the polymer with the corresponding morphology by controlling the reaction conditions. Common templates usually have porous membrane materials, fibers, colloidal particles and so on. This method is mainly used to prepare PPy hollow particles, NTs and NWs materials.

Martin et al. [37] reported the fabrication of PPy tubes using anodized aluminum oxide (AAO) as a template for the first time, but the diameter of the resulting tubes was on the micrometer scale, and template removal was difficult. With the progress of technology, the method is also improving. Sulkaa et al. [81] successfully fabricated hydroquinone monosulfonate-doped PPy NW arrays in AAO membranes with an aperture of 80 nm using a potentiostatic method and used them as potentiometric pH sensors. It was demonstrated that the pH sensor based on PPy nanowires has better electrochemical performance than that of PPy film.

V_2_O_5_ is also a common hard template for preparing PPy nanomaterials [108,109,110,111,112]. Zhang et al. [109] prepared PPy NTs with a pore size of less than 10 nm by using FeCl_3_ as an oxidant and V_2_O_5_ nanofibers (NFs) as a sacrificial template. Zhao et al. [112] prepared encapsulated PPy hollow nanowires by in situ polymerizations of pyrrole using V_2_O_5_ as a hard template. The hollow nanowires show a remarkably high adsorption capacity of 839.3 mg g^−1^ for 200 ppm Cr (VI) at pH = 2.

In addition, it has been reported that 1D PPy nanostructures can also be obtained by using TiO_2_ [113,114], MnO_2_ [115], and Fe_2_O_3_ [116] with different morphologies as sacrificial templates.

### 3.3. Templateless Method

The templateless method is to control the diffusion of monomers and oxidants in two incompatible phases and control the polymerization reaction conditions by means of interface action so that the polymer can be self-assembled into tubes, spheres, films and other special morphologies by using weak interactions such as hydrogen bonds, electrostatic interactions and coordination bonds between molecules. Template-free methods include electrochemical control [117,118,119,120,121,122], lithography [123,124,125], radiation [126,127] and others [128,129,130]. Because its preparation process is simple and does not require a specific sacrificial template, it has been widely studied for the preparation of PPy nanomaterials [131,132].

**Figure 6:**
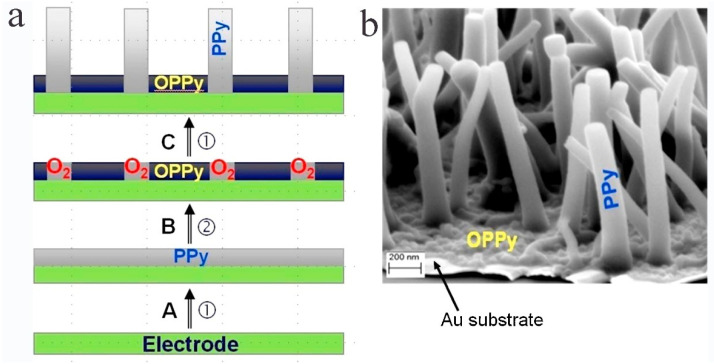
(**a**): Schematic model showing the process of PPy nanowire electrogeneration under potentiostatic conditions. Step A: Electrodeposition of an ultra thin PPy film. Step B: Generation of OH**·** which overoxidize PPy (OPPy) and of O_2_ nanobubbles which protect PPy against the action of OH**·**. Step C: Growth of the PPy nanowires. Reaction (1): Py oxidation; Reaction (2): water oxidation. (**b**): SEM micrograph of PPy deposited on Au/mica substrate [121]. Copyright 2015 Electrochimica Acta.

Using a constant current method, Wang et al. [117] prepared micro/nanoscale highly electroactive PPy with a hollow “horn”-like structure (h-PPy) in a p-toluenesulfonate alkaline solution without any templates. The h-PPy has a high specific surface area, particularly good molecular chain order, and a large conjugation length, which contributes to improving ionic and electronic conductivity. Fakhry et al. [121] deposited an ultrathin nonconductive peroxide PPy film on the electrode by the anodic polarization method in an atmosphere of a high concentration of weak acid anions without the help of any template (Figure 6), resulting in an aqueous solution of pyrrole with a pH of approximately 9. By introducing an advanced and simple electrohydrodynamic lithography (EHL) technique, Rickard et al. [123] patterned conductive polymers (CPs) directly on a high-fidelity substrate. They constructed thin PPy membranes through field-induced instability, resulting in well-defined conductive structures with feature sizes in the range of hundreds of nanometers to tens of microns, thus demonstrating the universality of this robust, low-cost approach. Furthermore, Cui et al. [127] succeeded in developing a new γ-radiolysis-based alternative method for synthesizing spherical and chaplet-like PPy nanostructures in solution.

In this section, we classify and discuss the different synthesis methods of PPy nanomaterials. Table 2 summarizes the relevant references classifying preparation methods, morphology and properties of PPy nanomaterials.

## 4. Application of PPy Nanomaterials

PPy nanomaterials have potential applications in energy storage, biomedicine, sensors, and other fields due to their excellent electrical, optical, and biological properties.

### 4.1. Energy Storage

CPs materials are lightweight, low-priced, and have good environmental compatibility. The maximum storage capacity of energy storage devices such as capacitors and batteries can be greatly enhanced by modifying the traditional positive or negative electrodes with CPs materials [133,134,135,136]. For energy storage devices, electrode material is the most critical factor affecting the performance of the entire capacitor, which determines its power, energy density and service life. The control of morphology, size and texture of electrode materials with higher power density, faster charge/discharge rate and longer-term stability is very interesting and especially important. PPy nanomaterials can be used as electrodes of energy storage devices, so the preparation of PPy nanomaterials is the key to fabricating energy storage devices. The preparation of PPy nanomaterials has been relatively mature, and the required size and morphology of nanomaterials can be prepared by the methods described above, so that the electrode materials with outstanding performance can be obtained.

#### 4.1.1. Battery

PPy nanomaterials in the field of batteries has mainly focused on three aspects: dye-sensitized solar cells [137,138,139,140], lithium and sodium batteries [34,141,142], and fuel cells [143,144,145,146].

Using SDS as a template, Hwang et al. [138] synthesized ultrathin PPy nanosheets (UPNSs) through organic single crystals surface-induced chemical oxidation polymerization. The power conversion efficiency of dye-sensitized solar cells (DSSC) using HCl-enhanced UPNS counter electrode was 6.8% (100 mW cm^−2^). This result is 19.3% higher than that of the untreated condition and comparable to DSSCs using Pt as a counter electrode. Wen’s team [141,142] synthesized highly ordered PPy NTs for lithium-sulfur batteries. Lithium-sulfur battery with the PPy NTs exhibits an inspiring electrochemical property. Sun’s team [144,145] developed a simple electrochemical polymerization for the preparation of PPy NWs on Pd modified Nafion^®^ membranes. The ordered PPy NWs can significantly improve fuel cell performance by facilitating mass transfer and enhancing catalyst utilization.

#### 4.1.2. Supercapacitor

**Figure 7:**
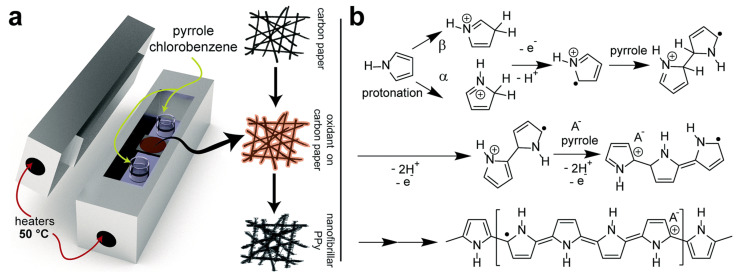
(**a**) A schematic of evaporative vapor phase polymerization, (**b**) An acid-catalyzed chain-growth mechanism of polymerization for pyrrole [155]. Copyright 2017 Journal of Materials Chemistry A.

In the field of supercapacitors, CP-based supercapacitors have received increasing attention due to their large specific electric capacity. However, they have poor stability, and researchers have attempted unremittingly to enhance the electrical performance and stability of PPy-based supercapacitors [61,82,97,100,115,117,147,148,149,150,151,152,153,154,155,156,157,158,159] (see Table 3). Santino et al. [155] coated a high-aspect ratio bristle-like nano-PPy continuous network on a graphitic hard carbon paper current collector through a modified gas phase polymerization. Nano-PPy based electrodes exhibit good performance at high discharge rates, as shown in Figure 7.

There are abundant heteroatoms in the conductive polymer skeleton, which can be uniformly dispersed in the carbon skeleton in situ after carbonization, obtaining heteroatom doped carbon nanotubes with excellent physical and chemical properties. Carbon nanotubes derived from conductive polymers show a good application prospect in the fields of supercapacitor. Shen et al. [158] developed a simple approach to fabricating uniform PPy nanospheres using 3-chloroperbenzoic acid as an oxidant, dopant and structural guiding agent, then pyrolyzed the PPy nanospheres at 900 °C to form nitrogen-doped carbon nanospheres, which exhibited good conductivity, excellent electrochemical properties and good stability.

In addition to being used as traditional supercapacitors, PPy nanomaterials have recently been reported for flexible supercapacitor applications [160,161,162]. Shi et al. [160] reported a simple and versatile synthesis approach to using PPy hydrogels with tunable 3D microstructures as electrically active materials for flexible solid-state supercapacitors with high performance. The supercapacitors fabricated on the basis of the flexible symmetric PPy hydrogels exhibited good capacitive performance and electrochemical stability during long-term cycling.

**Table 3:** A summary of the morphologies of PPy and the capacitive properties of PPy-based supercapacitors.

Morphology	Configuration	Capacitance	Cyclability	Ref.
Nanowire arrays	Symmetric capacitors	699 F g^−1^ (1 A g^−1^)	63% (5000 cycles, 50 A g^−1^)	[82]
Nanochains	Single electrode	1502 F g^−1^ (2 mV s^−1^)	93% (1500 cycles, 1 A g^−1^)	[100]
Nanowires	Single electrode	328.7 F g^−1^ (0.3 A g^−1^)	75.7% (600 cycles, 1.5 A g^−1^)	[107]
Nanofibers	Single electrode	604 F g^−1^ (1.81 A g^−1^)	91% (1000 cycles, 9 A g^−1^)	[115]
Hollow “horns” in micro/nanometers	Single electrode	400 F g^−1^ (3 A g^−1^)	90% (100,000 cycles, 500 mV s^−1^)	[117]
Nanobricks	Single electrode	476 F g^−1^ (5 mV s^−1^)		[149]
Nanoplates	Single electrode	533 F g^−1^ (5 mV s^−1^)	78% (5000 cycles, 100 mV s^−1^)	[150]
Nanosheets	Single electrode	586 F g^−1^ (2 mV s^−1^)	81% (5000 cycles, 100 mV s^−1^)	[152]
The clusters of nanofibers and nanoparticles	Single electrode	427 F g^−1^ (0.02 A cm^−1^)		[153]
Nanobrushes	Symmetric capacitors	144.7 F g^−1^ (20 mV s^−1^)	70% (20,000 cycles, 5 A g^−1^)	[155]
Nanowires	Single electrode	420 F g^−1^ (1.5 A g^−1^)	97.9 % (8000 cycles, 1.5 A g^−1^)	[156]
Films with hollow micro/nano-scaled horn arrays	Single electrode	360 F g^−1^ (10 mV s^−1^)	88.2% (10,000 cycles, 30 A g^−1^)	[157]
Nanospheres	Single electrode	176 F g^−1^ (1 A g^−1^)		[158]
Films with Micro/Nanosphere Shapes	Single electrode	568 F g^−1^ (20 mV s^−1^)	77% (10,000 cycles, 10 A g^−1^)	[159]
Hydrogels	Symmetric capacitors	380 F g^−1^ (0.2 A g^−1^)	90% (3000 cycles, 100 mV s^−1^)	[160]
3D interconnected fibrous structure	Symmetric capacitors	168 F g^−1^ (2 mA cm^−2^)	97% (2000 cycles, 10 mV s^−1^)	[162]

### 4.2. Biomedicine

Compared with bulk CP materials, as well as ceramic and metal nanomaterials, CP nanomaterials have outstanding physicochemical properties [29,163,164,165]. Nano-PPy is an intriguing candidate in biomedical applications due to its unique properties among CPs [166].

#### 4.2.1. Drug Delivery and Release

CPs can undergo reversible electrochemical reactions, with volume shrinkage during reduction and volume expansion after oxidation, which is beneficial for the controlled release of various drugs [167,168,169,170,171,172,173,174,175,176,177]. PPy nanomaterials have the advantages of easy drug loading, having little effect on drug activity, and a controllable drug release rate. Samanta et al. [170] synthesized PPy NPs that were stably dispersed in solution and had good drug loading capacity (15 wt%) through a simple microemulsion polymerization technique. The prepared PPy NPs can be adjusted to release drugs by changing the pH, the charge of the drug, and adding a small amount of charged amphiphiles. In order to provide high loadings of hydrophobic drugs, Moquin et al. [176] obtained linear hydrophobic pyrrole-based polymers with attached hydrophilic polyethylene glycol chains by one-pot coupling of diimine, terephthaloyl chloride and substituted alkynes. The amphiphilic PEGylated PPy can easily self-assemble into soft NPs.

#### 4.2.2. Photoacoustic and Photothermal Therapy

**Figure 8:**
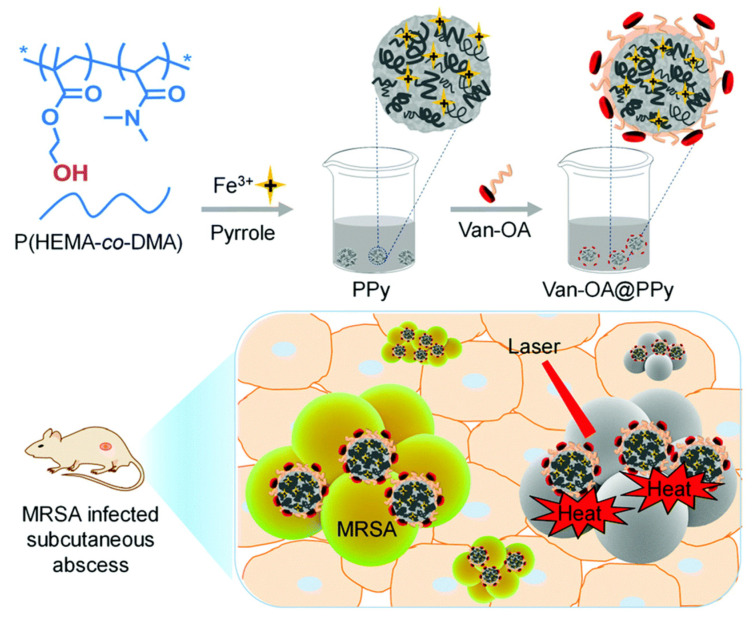
Schematic illustration for in situ formulation of pathogen-targeting phototheranostic nanoparticles, Van-OA@PPy, for photothermal inhibition of MRSA infection. P(HEMA-co-DMA) was employed as a template to afford PPy in situ in the presence of Fe^3+^, and then PPy was further self-assembled with vancomycin-tethered oleic acid (Van-OA) to afford the resultant phototheranostic Van-OA@PPy. [183]. Copyright 2020 Nanoscale.

PPy nanomaterials has good biocompatibility, outstanding photostability and photothermal conversion properties, which has broad application prospects in the field of photothermal therapy. Among PPy nanomaterials, PPy NPs are the first and most widely used in photothermal therapy [178,179,180,181,182,183,184,185,186]. Yang et al. [180] prepared biocompatible PPy-PVA core-shell NPs for the photothermal elimination of tumors in vitro and in vivo at ultralow laser power density. The composite NPs were injected intratumorally and further irradiated with a 0.25 W cm^−2^ power near-infrared laser, a good tumor therapeutic effect was achieved and no obvious side effects were observed. Later, Dai’s team [181,182] prepared homogeneous PPy NPs by a simple chemical oxidation polymerization method. Owing to strong near-infrared absorption and good photostability, the as-prepared colloidal-stabilized PPy NPs exhibited remarkable photothermal conversion efficiency. In order to obtain materials with high photothermal conversion efficiency, Guo et al. prepared PPy NPs using hydrophilic poly (2-hydroxyethyl methacrylate-co-N, N-dimethyl acrylamide), P(HEMA-co-DMA) as a template and Fe^3+^ as an oxidant. The prepared PPy NPs are further encapsulated by vancomycin conjugated oleic acid (Van-OA) to provide final pathogen targeting Van-OA@PPy, which exhibited a high photothermal conversion efficiency of ~49.4%. The preparation process and photothermal conversion mechanism of Van-OA@PPy are shown in Figure 8.

The application of hollow PPy nanomaterials in the field of photothermal therapy has also been reported [187,188,189]. Bhattarai et al. [188] prepared PPy hollow fibers by polymerization of pyrrole on the sacrificial templates of electrospun polycaprolactone fibers. The results show that the initial concentration of pyrrole, near-infrared laser power and irradiation time are the key factors affecting their photothermal performance. Compared with the PPy-NPs counterpart, the manufactured PPy hollow fibers exhibit enhanced photothermal performance.

In addition to the above mentioned nanostructured PPy, researchers found that PPy nanosheets also have positive effects on photothermal therapy [190,191]. Wang’s team [190] fabricated 2D ultrathin PPy nanosheets via a space-constrained approach. The as-prepared PPy nanosheets showed special broadband absorption at 1064 nm and had a large extinction coefficient of 27.8 L g^−1^ cm^−1^, which could be applied as an effective photothermal agent in the second near-infrared window.

### 4.3. Sensors

In the field of sensor applications, it is very important to increase the sensitivity and reduce the operating temperature. CP-nanomaterials based sensors have the advantages of low price, high sensitivity, detecting diversity and fast response speed. The sensors prepared from PPy nanomaterials are mainly used for biological and chemical detection.

#### 4.3.1. Biosensors

PPy nanomaterials have been used in the fabrication of various biosensors due to their unique properties [192,193]. Biosensors mainly detect proteins [194,195,196], hormones [197,198], DNA [199,200,201], RNA [202,203], Cu^2+^ [204,205] and others [206,207,208,209,210].

Field effect transistor (FET) is a kind of transistor that uses electric field to control the conductivity of charge carriers in semiconductor materials. The FET based biosensor device uses its current amplification characteristics to improve its measurement accuracy, thus increasing the detection possibility of low concentration analytes. FET-type sensors based on PPy nanomaterials can be used to detect proteins used as cancer suppressors or markers. In order to fabricate PPy-based FET sensors to detect bioactive factor, Jang’s team carried out the following:

1. Introduced amino groups on an interdigitated microelectrode array (IDA) substrate;
2. Immobilized carboxylated PPy nanomaterials on IDA substrate to maintain stable electrical contact between the PPy and the microelectrodes;
3. Attached the aptamer to the surface of carboxylated PPy nanomaterials by coupling reaction.
4. Acting as the grid dielectric of the p-type FET sensor, the target molecule specifically interacted with the adapter attached to the PPy surface.

**Figure 9:**
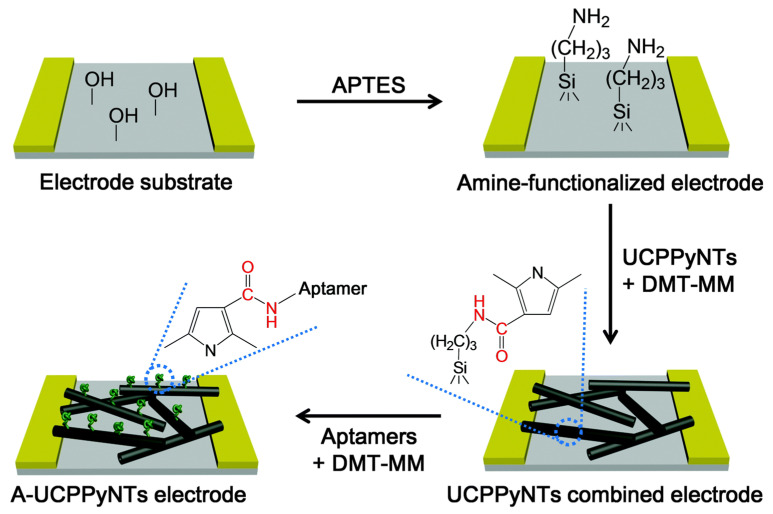
Schematic diagram of the binding process used to produce the biosensor electrode based on A-UCPPyNTs on the IDA electrode substrate [198]. Copyright 2016 Journal of Materials Chemistry B.

Through the above means, his team [194] developed a speedy and effective technique for detecting a novel heat shock protein 90 inhibitor as an anticancer medicine using a FET sensor based on carboxypolypyrrole nanotubes (CPNTs). In addition, his team used a p-type FET biosensor to detect vascular endothelial growth factor (VEGF) as a cancer biomarker in vitro electrochemical detection [195]. A high-performance FET sensor based on an anti-VEGF RNA aptamer combined with CPNTs can detect VEGF concentrations as low as ca. 400 fM. Furthermore, his team [197,198] prepared carboxylated PPy NPs and NTs (Figure 9) for the detection of various hormones, such as peptide hormone and 17 beta-estradiol.

The detection of DNA and RNA is also commonly used in biosensors based on PPy. PPy nanomaterials modified with redox markers are a promising platform for electrochemical biosensors, which can be applied to various diagnostic prospects. Without using any templates, Khoder et al. [201] prepared PPy NWs using simple electropolymerization. Without any amplification operations, the detection limit of the PPy NWs modified E-DNA biosensor with ferrocene as the redox marker is 0.36 aM. Wang et al. [203] developed an ultrasensitive biosensor based on PEG-PPy NWs substrate using a simple electrochemical model strategy to provide better antifouling performance. The developed biosensor has good selectivity and can effectively identify miRNA mismatches.

Heavy metals have caused great harm to the environment and human health. The biosensors developed by Lin et al. [204,205] include carboxyl end-capped peroxidized PPy NW/NT electrodes and tripeptide (Gly-Gly-His) probes for the selective recognition of Cu^2+^.

#### 4.3.2. Chemical Sensors

Chemical sensors are commonly used for the detection of NH_3_ [33,87,89,92,211,212,213,214,215,216,217,218], volatile organic compounds [31,106,219,220,221], variation in pH values [81,109,222] and others [223,224,225,226,227,228] (see Table 4).

The researchers compared the performance of PPy nanomaterials and bulk PPy for ammonia sensors. It was found that the sensors made of PPy nanomaterials perform better than those made of bulk PPy materials, and the sensors made of PPy nanomaterials with larger specific surface area have better properties. Yang et al. [211] synthesized homogeneous PPy NFs with high yield using FeCl_3_ as an oxidant and MO as a template. Compared with the sensors based on bulk PPy and PPy NPs, the NH_3_ sensors based on PPy NFs showed a remarkably enhanced performance. Rawal et al. [216] obtained PPy NWs in the existence of MO, while PPy NPs were prepared under similar conditions with the aid of CTAB. Compared with PPy NPs, PPy NWs were found to have a higher doping, bipolaron concentration, porosity, and conductivity. Meanwhile, the PPy NWs-based sensors exhibited better sensitivity than the PPy NPs-based sensors.

The technology of detecting volatile organic compounds (VOCs, such as acetone, ethanol, acetic acid, etc.) with high sensitivity and a fast response time has many potential applications in home health care, work automation and disaster prevention. Sensors based on PPy nanomaterials are also commonly used to detect VOCs. Alizadeh et al. [219] prepared nanostructured conductive PPy on interdigital electrodes by galvanostatic electrodeposition under anion doping. Sensors based on anion-doped PPy have been confirmed to have a response time of less than 1 s, high selectivity and calibration sensitivity, and good reproducibility for methanol at room temperature.

Sensors based on PPy nanomaterials are also used for pH monitoring. Shirale et al. [222] synthesized PPy NWs with different diameters using electrochemical deposition inside AAO templates and investigated the effects of different aspect ratios on real-time pH monitoring on FET sensors based on single PPy nanowires. These single PPy nanowire-based FET sensors exhibited excellent and adjustable sensitivity to pH changes and recorded higher sensitivity with a higher aspect ratio of the PPy nanowire.

### 4.4. Others

#### 4.4.1. Absorption and Impurity Removal

PPy nanomaterials have received remarkable attention in the area of adsorption and impurity removal owing to ease of preparation, and environmentally friendly and excellent oxidation-reduction properties. Usually, PPy nanomaterials are used to absorb heavy metal ions [110,112,229,230,231,232] and organic pollutants [233,234].

Because heavy metals have high solubility in water and are widely used in different industries, removing heavy metals from water in an efficient manner has become a global challenge. Among many heavy metals, chromium (VI) is harmful and widespread. Due to the nitrogen-containing structure of pyrrole, PPy exhibits excellent adsorption capacity for Cr (VI). Zhan et al. [110] reported a reusable bamboo-like PPy nanofiber mat for Cr (VI) adsorption. At pH = 2, the flexible bamboo-like PPy nanofiber mat has a high adsorption capacity of 961.5 mg g^−1^ for Cr (VI). Using Fe_3_O_4_ nanoclusters as a template and oxidant, Yao et al. [229] prepared hierarchical porous PPy nanoclusters with a larger specific surface area and higher conductivity in a one-step method. The as-prepared PPy nanoclusters showed an outstanding capacity for removing Cr (VI) compared to active carbon and PPy NPs.

The organic dyes in wastewater are harmful to human beings and the environment, so it is important to remove them effectively. Xin et al. [234] reported an adsorbent of PPy NFs for removing MO from an aqueous solution. The adsorption capacity of MO by PPy NFs can reach 169.55 mg g^−1^ at 25 °C.

#### 4.4.2. Wave-Absorbing Materials

As a conductive loss-absorbing material, PPy will generate an induced electric field when it is induced by an external magnetic field, and the induced electric field will generate an induced magnetic field opposite to the external magnetic field, thereby absorbing and shielding electromagnetic waves [76]. Wong et al. [235] first used the large-scaled PPy doped with toluenesulfonate anion in the electromagnetic shielding field. However, the preparation of PPy materials is not convenient in experiments and requires a long processing time. The preparation of PPy nanomaterial is convenient and the electromagnetic shielding effect is better. Kaur et al. [236] synthesized PPy NPs by a surfactant-directed chemical oxidation method. It is found that the particle size of the PPy NPs decreases while the dc conductivity and total shielding effectiveness increase with surfactant concentration in the reaction solution. Xie et al. [237] synthesized one-arm helical PPy nanostructures through the chirality induction route. Due to the gradually constructed conductive network and spiral chirality, it has tunable and impressive electromagnetic protection performance.

#### 4.4.3. Solid Phase Extraction

Solid phase extraction (SPE) is currently the most widely used sample pretreatment technology. In recent years, the miniaturization of SPE technology, especially the combination of SPE technology and fast-developing nanomaterials to achieve efficient and rapid sample pretreatment has become a research focus. Wu and others in the research group of Pawliszyn, the founder of SPE, have performed a series of studies on the extraction separation use of PPy [238,239,240]. Based on previous work, the applications of PPy nanomaterials in SPE have made progress [241,242,243,244,245,246]. Lazzari et al. [245] reported a modified electrode coated with PPy NTs on the surface of a steel mesh as an adsorption phase, which can easily, quickly and inexpensively extract atrazine, progesterone, and caffeine from aqueous solutions. Due to the larger surface area and lower relative standard deviation (RSD) value, PPy NTs improved the extraction efficiency and showed better performance for adsorption phenomena. Xie et al. [246] proposed a prospective fiber-filled SPE precleaning approach that uses PPy electrospun NFs as adsorbents to simultaneously extract three water-soluble vitamins from human urine.

#### 4.4.4. Actuators

PPy is characterized by its small mass, soft plastids, ease of processing, good biocompatibility, large electrostrain (bending or stretching), and ability to work in the air and liquid media. Under voltage stimulation, a reversible oxidation-reduction reaction will occur inside it, causing changes in volume and mechanical properties, and it can return to its original shape or volume after the voltage excitation is removed. Therefore, it can be used as an actuator. This kind of material can be miniaturized for component design, making it a microelectronic mechanical system device that is widely used in microrobots, microvalves, biomedical electronic devices and other fields [247,248,249,250,251,252].

**Figure 10:**
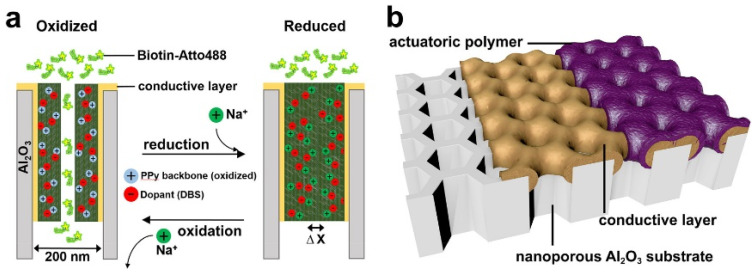
(**a**) Scheme of the electrochemical based opening and closing of the nanovalves, (**b**) Layer composition on the nanoporous aluminum oxide substrate [248]. Copyright 2018 Nano Letters.

According to the mechanism that allows PPy to change its volume according to the redox state, Christoph et al. [248] proposed a novel electrically tunable nanovalve array based on nanostructured PPy that has a naturally open state when no potential is applied and can be closed when a reduction potential is applied, as shown in Figure 10. It was found that the PPy layer doped with DBS shows a driving performance of up to 10% of the planar volume change. During the oxidative fabricating process of PPy, the positively charged framework is introduced on the conductive surface of porous noble-metal substrate. The flow and integration of doped anions that fix in polymer matrix balance the charge, thus creating an opened nanovalve. In the reduction process of PPy, the reduction potential is applied to neutralize the polymer chain. Cations around the electrolyte transfer to the anions fixed in polymer to achieve charge balance. This process is accompanied by osmotic pressure, leading to the incorporation of water, which causes the expansion of polymer and finally closes the nanovalve.

Taking advantage of the hydrophobicity of implants and the conductivity of PPy, Liao et al. [250] incorporated the amphiphilic biomolecular taurocholic acid (TCA) into the 1D nanostructured PPy array for implants. TCA plays an important role in the fabrication of this device. Within a suitable concentration range, it is used as a surfactant to conduct the self-assembly of pyrrole micelles on biomedical titanium implants to form a tapered 1D nanostructured PPy. In order to respond the periodic switching of potential, TCA molecules will change the orientation of their hydrophobic and hydrophilic surfaces in PPy matrix. The implant surface doped with TCA showed reversible wettability between the open state of 152° (superhydrophobic) and the closed state of 55° (hydrophilic) in response to switching on and off power potentials periodically between the two potentials of + 0.50 and −0.80 V.

In addition to the application fields investigated above, the application of PPy-based nanomaterials in other aspects has also been reported, such as the moistelectric nanogenerator [75], corrosion protection [253,254], hydrogen evolution [255], thermoelectric [256], chemical mapping [257] and ink formulations [258].

## 5. Conclusions and Perspectives

PPy nanomaterials show high conductivity, large surface area and many other properties. As described in this paper, many innovative fabrication methods have been developed for the preparation of spherical, tubular, wire, rod, sheet, helical and other PPy nanomaterials, including electrochemical polymerization, interfacial synthesis, emulsion polymerization, radiation-initiated polymerization, surfactant-assisted polymerization, gas-phase polymerization, electrospinning, etc. Functional PPy nanomaterials prepared by these means display many attractive properties, which have been extensively explored in the applications of energy storage, biomedicine, sensors, adsorption and impurity removal, microwave absorption, solid-phase extraction and actuators.

Although research on PPy nanomaterials is progressing rapidly, there are still many tasks to be completed. Firstly, precisely controlling the size and morphology of PPy nanomaterials is still a major challenge in this field. By accurately controlling the size and morphology of PPy nanomaterials, a series of materials with excellent optical, thermal and electrical properties can be obtained, which broaden the application of PPy. Therefore, future developments should focus on improving synthetic methods and deriving novel assembly processes for better control of the size and structure. Secondly, the accuracy of characterization is expected to improve, while the repeatability also needs to be improved, which has a certain impact on the study of the mechanism. Lastly, there are still many important problems in the application of PPy nanomaterials, and few can be used in commercial applications. In order to realize commercial application as soon as possible, the following is necessary: the environmental stability of PPy nanomaterials needs to be improved, the new environmentally friendly PPy nanomaterials need to be developed, and the application fields of PPy nanomaterials need to be further expanded. It is foreseeable that combining another suitable component with PPy nanomaterials will be a very promising material for various applications. However, there is still a need to study new methods of preparing this material, discover interesting and enhanced properties, and expand its applications.

## Figures and Tables

**Table 1:** A summary of synthetic conditions, size and conductivity of PPy nanoparticles.

Morphology	Surfactant/Template	Dopant	Oxidant	Diameter (nm)	Conductivity (25 °C, S/cm)	Ref.
Spheres	Castor oil sulfate	Castor oil sulfate	(NH_4_)_2_S_2_O_8_	20–100	1–6	[7]
Spheres	PVP		FeCl_3_	30–60	10–15	[8]
Particles	CTAB	HCl	(NH_4_)_2_S_2_O_8_	20		[9]
Particles	Polyoxyethylene nonylphenyl ether sulfate	Polyoxyethylene nonylphenyl ether sulfate	(NH_4_)_2_S_2_O_8_	40–150	1–20	[10]
Particles	SDS		FeCl_3_	28–52	3–22	[11]
Spheres	Fatty alcohol–polyoxyethylene ethers		(NH_4_)_2_S_2_O_8_	60–230		[12]
Particles	Sodium taurocholate and Tween 20	Citric acid	H_2_O_2_	100–500	10^−2^	[13]
Spheres		Iodine		35–350	10^−7^–10^−4^	[14]
Spheres		Heparin		50–80		[17]
Irregular	Methyl red	Methyl red	FeCl_3_		84	[19]
Spheres	Pluronics^®^ F-108	Formic acid	(NH_4_)_2_S_2_O_8_	110–120		[21]
Particles	PVA		FeCl_3_	20–60	0–10	[23]
Particles	Poly (ethylene glycol) (PEG)	Organic sulfonic acid	(NH_4_)_2_S_2_O_8_	71–134	3.26–52.7	[24]
Particles	SDS	HCl	H_2_O_2_	28		[25]
Particles	PVP	H_2_SO_4_	H_2_O_2_	21–92	7.67 × 10^−3^	[26]
Particles	PVA		FeCl_3_	20–100		[28]
Particles	PVA		FeCl_3_	20–80	1.71–149.08	[30]
Particles	PVA		FeCl_3_	20–100	1–10	[31]
Double-walled shells	Au nanocages	PVP	FeCl_3_	5 nm (two shells spacing)		[32]
Urchin-like particles		PVA	FeCl_3_	30, 60, 100		[33]
Hollow spheres	Poly (methyl methacrylate)		FeCl_3_	136.5 (inner) and 242 (outer)		[34]
Double-shelled hollow particles	Polystyrene	PVP	FeCl_3_			[35]
Bowl-shaped particles		Iodine	FeCl_3_			[36]

**Table 2:** A summary of preparation methods, morphology and properties of PPy nanomaterials.

Method	Morphology	Reaction Medium	Oxidant	Properties	Ref.
Hard template/Electropolymerization	Nanowire arrays	A 0.1 M LiClO_4_ solution		A good potentiometric response to pH changes and a very good stability in time	[81]
Soft template/ Chemical oxidation	Nanoscale hierarchical structure	Aqueous solution	FeCl_3_	A high specific capacitance and good electrochemical reversibility	[104]
Soft template/Electropolymerization	Nanostructured membranes	Milli Q water		A high specific surface area and high specific capacitance	[105]
Soft template/Chemical oxidation	Nanoparticles	Aqueous solution	FeCl_3_	Potentially useful to detect acetone	[106]
Soft template/Interfacial polymerization	Nanowires	Organic/aqueous interface	(NH_4_)_2_S_2_O_8_	A high specific capacitance	[107]
Hard template/Chemical oxidation	Nanotubes	Ethanol solution	FeCl_3_	Undergo a spontaneous redox reaction with metal ions	[109]
Hard template/Chemical oxidation	Nanofibers	Aqueous solution	H_2_O_2_	Bulk quantities	[111]
Hard template/ In-situ vapor phase polymerization	Hollow nanofibers	In desiccators		A high Cr (VI) adsorption capacity up to 839.3 mg g^−1^	[112]
Hard template/Electropolymerization	Nanopore arrays	An ionic-surfactant-solution		Forming mechanically stable and underlying compact films	[113]
Hard template/Electropolymerization	Nanowires and nanopore arrays	Electrolyte of dodecyl sulfate		Well-organized and mechanically stable	[114]
Hard template/Chemical oxidation	Nanofibers	Aqueous solution	K_2_Cr_2_O_7_	Enhanced electroactive surface area	[115]
Hard template/Electropolymerization	Nanotube arrays	CH_2_Cl_2_ or acetonitrile solution		A facile, inexpensive and large-scale means for generating polymeric nanostructures	[116]
Template-free/Electropolymerization	Hollow “horns” in nanometers	P-toluenesulfonate alkaline solution		High specific surface area and high ionic and electronic conductivity	[117]
Template-free/Electropolymerization	Nanotube arrays	Phosphate buffer solution		Enhanced electrical and electrochemical performances	[118]
Template-free/Electropolymerization	Nano-snails	Aqueous alkaline solution	Fe (CN)_6_^3−^	Promising potential applications in supercapacitors and sensors	[120]
Template-free/electropolymerization	Nanowires	A 70:30 H_2_O/EtOH mixture		Forming a uniform polymer film	[122]
Template-free/electrohydrodynamic lithography	Nanostructured films	Aqueous solution	(NH_4_)_2_S_2_O_8_	Accessing scale sizes in the low submicron range	[123]
Template-free/electrochemical lithography	Nanostructured films	Aqueous solution		A reversible, erasable, and rewritable pattern	[124]
Template-free/edge nanoimprint lithography	Nanowires	Aqueous solution	FeCl_3_	Exhibiting representative ohmic behavior and excellent sensitivity to NH_3_	[125]
Template-free/γ-radiation-induced chemical oxidative	Polydisperse spherical nanoparticles	Aqueous solution	K_2_S_2_O_8_	Well dispersed in water, easily dried and quite simply redispersed in protic solvents	[127]
Template-free/mechanochemical route	Nanospheres	Pre-cleaned mortar	K_2_S_2_O_8_	High degree of processability, electrochemical activity and film forming ability	[128]
Template-free/chemical oxidation	Nanospheres	Aqueous solution	O_3_	Stable and unagglomerated	[129]
Template-free/electropolymerization	Nanowires	Acetonitrile solution	Fe (CN)_6_^3−^	Low cost, simplicity, rapidity, and versatility	[131]

**Table 4:** A summary of PPy nanomaterials for sensors.

Morphology	Analyte	Linear Range	Detection Limit	Ref.
Nanoparticles	Acetone	5.5–80 ppm	5.5 ppm	[106]
Nanotubes	Heat shock protein 90 inhibitors	40 nM–8 μM	40 nM	[194]
Nanotubes	Vascular Endothelial Growth Factor	400 fM–4 μM	400 fM	[195]
Nanowires	IgE protein	0.01–100 nM	0.01 nM	[196]
Nanoparticles	Peptide hormones	48 fM–48 pM	48 fM	[197]
Nanotubes	17β-estradiol	1 fM–1 nM	1 fM	[198]
Nanowires	DNA	10 pM–500 nM	10 pM	[199]
Nanowires	Escherichia coli DNA		0.1 nM	[200]
Nanowires	DNA	1 aM–100 fM	0.36 aM	[201]
Nanowires	microRNA	0.1 pM–1 nM	0.033 pM	[203]
Nanowires	Cu^2+^	20–300 nM	20 nM	[204]
Nanotube arrays	Cu^2+^	0.1–30 μM	46 nM	[205]
Nanoribbons	Viral plant pathogen	10 ng ml^−1^–100 μg ml^−1^	10 ng ml^−1^	[206]
Films	SARS-CoV-2-S glycoprotein	0–25 μg ml^−1^	0.15 μg ml^−1^	[207]
Nanotube arrays	Glucose	0.2–13 mM	50 mu M	[208]
Nanoparticles	H_2_O_2_	5–100 μM	5 μM	[209]
Nanorods	Nitrate	1.0 × 10^−4^–5.0 × 10^−3^ mol L^−1^	5.0 × 10^−5^ mol L^−1^	[210]
Nanotubes	NH_3_		0.01 ppm	[212]
Nanowires	NH_3_	1–100 ppm	0.4 ppm	[213]
Nano-dumbbells	NH_3_	1 ppb–1 ppm	1 ppb	[217]
Nanoparticles	NH_3_, acetic acid	1–100 ppm	0.1 ppm, 1 ppm	[221]
Nanowires	H_2_	600–2500 ppm	12 ppm	[223]
Nanonecklaces	2,4-dichlorophenoxyacetic acid	0.1–8 μM	100 nM	[224]
Nanobelts	Methanol	20 μM–0.16 mM	6.92 μM	[225]
Nanoparticles	Pb^2+^	0.1–50 μM	55 nM	[226]
Nanoparticles	Bisphenol A	1–10^4^ fM	1 f M	[228]

## Data Availability

Data presented in this study are available on request from the corresponding author.
